# Compatibility of Conservative and Aggressive Surgery

**Published:** 1892-12

**Authors:** J. McFadden Gaston

**Affiliations:** President; Atlanta, Ga.


ATLANTA
### MEDICAL AND SURGICAL JOURNAL.

VOL. IX. DECEMBER, 1892. No. 10.

EDITED BY
LUTHER B. GRANDY, M. D., AND MILLER B. HUTCHINS, M. D.,
WITH THE CO-OPERATION OF
H. V. M. MILLER, M. D., LL. D., VIRGIL O. HARDON, M. D., AND
FLOYD W. McRAE, M. D.

### *ORIGINAL COMMUNICATIONS.*

### COMPATIBILITY OF CONSERVATIVE AND AGGRESSIVE SURGERY.

ANNUAL ADDRESS BEFORE THE SOUTHERN SURGICAL AND GYNECOLOGICAL ASSOCIATION, LOUISVILLE, KY., NOV. 16, 1892.

BY THE PRESIDENT, J. McFADDEN GASTON, M. D., ATLANTA, GA.

The circumspect philosophy of former days taught us, what man has done man may do. But the developments of more recent times say, whatever is practicable may be undertaken, without regard to precedents. Conservative and aggressive processes are combined in progressive surgery.

Conservatism in the use of all the appliances of surgery is not inconsistent with the application of the most energetic means of relief in structural disorders. A misapprehension exists with many of our profession as to the true sphere of progressive surgery, and it is my purpose on this occasion to make a distinction between rashness in the employment of operative measures and boldness in the use of surgical means of relief when clearly indicated. Real advances in surgical practice have not been the result of cutting and slashing without due consideration, but have accompanied painstaking investigation of the conditions requiring the knife, and caution in the performance of operations. As a preliminary to any surgical procedure of a radical nature, correct diagnosis is essential, but to accomplish a proper understanding of a deep seated disorder it is often requisite to make an exploratory operation of greater or less magnitude.

The information based upon such an exploratory measure serves as a guide to any further surgical procedure. A verification of any complication, of a serious nature, by an exploratory step, may form a contraindication for the more radical operation which has been contemplated. A great variety of means have been employed under the expectation of elucidating the doubts surrounding very obscure cases, some of which are of a precarious nature, proving far more hazardous than the disease or the proposed radical measure. It is evident that exploratory processes of this order should be ruled out of our surgical resources, and the prime consideration for our safe guidance in exploratory operations is to be sure that the existing disorders shall not be aggravated by such procedure, nor that other graver developments shall be induced as a consequence of it. It is best always in cases involving mooted points to avoid the appearance of evil. In keeping with this precaution against the abuse of exploratory measures, it is proper to exercise great discrimination in the use of antiseptics in surgery. A most salutary result may be obtained by employing very active germicides in a case of septic contamination, whereas the same application may not be suited to an operation upon tissues in their normal state. It is now recognized by bacteriologists that certain preparations tend to destroy pyogenic organisms, and when they exist the use of such antiseptic means is indicated. On the other hand, it is becoming fully understood by clinical experience that a resort to these germicidal agents, when the pyogenic organisms are not present, operates unfavorably upon the healthy structure. The reaction in the practice of many surgeons of large practical experience, from observing the absolutely hurtful effects of the ordinary solutions of corrosive sublimate, when brought in contact with the absorbent surfaces of normal structures, is tending to a limitation of this poisonous substance to external use. These do not belong to the class of "superfluous laggards" who are characterized as opposed to the methods of asepsis and antisepsis, by the author of a recent work on surgery. But they are of that independent order of creation who are not bound down by any prescribed formulas and are bold enough to abandon a vicious practice which has brought trouble in its train to many victims of routine treatment.

It is well known to all surgeons, who have investigated thoroughly the merits of antisepsis, that there are many germicidal applications far safer than corrosive sublimate, which meet all the requirements in surgery, and the day is not far distant when the use of the solutions of corrosive sublimate will be excluded from all operations upon incised healthy structures. There is a gradual movement of the pendulum of antisepsis towards asepsis, and it is found that sterilized water is the safest and best wash for surfaces not contaminated by previous septic developments in their tissues.

Antisepsis has a legitimate field of use in surgery, and it is to be regretted that the careless extravagance of the advocates of sublimate solutions should have given this process a black-eye by poisoning patients with them, who could have been treated successfully with other antiseptic agents.

Great detriment to proper antiseptic treatment has ensued from the indiscriminate application of solutions of corrosive sublimate, but judicious men are now more guarded in employing them in surgery.

Upon the general principle that no surgical operation of any magnitude should be undertaken without proper knowledge of the habits of life on the part of the patient, I may advert to a fact which is overlooked by many operators, in regard to the importance of continuing the use of any stimulant or narcotic to which the patient has long been accustomed. Within my experience the interruption even of the tobacco habit has been followed by troublesome nervous depression, after operations, and the resumption of the use of this narcotic has afforded complete and prompt relief. I recall an instance of laparotomy for the removal of an immense cystic tumor of the ovary in a lady sixty years of age who of her own accord stopped smoking the pipe after the operation. For a few days all seemed to be doing well with the case, but at the end of a week there was a most profound depression of spirits with vital prostration, which immediately disappeared upon resuming her pipe and the final result was entirely satisfactory.

We all know that a patient who has been addicted to the use of alcoholic drinks for a considerable period before submitting to any surgical operation cannot have it suddenly abstracted without being subjected to great disturbance of the entire nervous system. If he does not reach the point of perturbation to produce delirium tremens, there will still be such derangement of all the functions as to interfere materially with proper nutrition, which may pave the way to serious complications in the after treatment of the case. It is, hence, essential under such circumstances, to take into account the previous habit in this respect, and continue to give certain quantities of the alcoholic stimulant at fixed periods to avert any troublesome consequences. An individual may fail to give such information as to habits of life in regard to the use of alcoholic stimulants unless specially interrogated and yet would not hesitate to state the fact upon inquiry for the guidance of the surgeon in his case.

The most important matter, however, for our investigation is in regard to the opium habit in one subjected to a grave operation. I am convinced from a case which has recently been reported to me, that operators of experience and with a full knowledge of the use of morphine by a patient, do not always realize the great detriment resulting from the sudden withdrawal of this article after an important operation. All abnormal conditions resulting from any habit should be compensated in the after treatment. Observation of the changes resulting from inflammatory processes, should be accompanied by a study of those modifications impressed upon the tissues by impairment or undue activity of the nerve element, which enters into their composition. That many operators fail to take into account the nervous system, in their surgical pathology, shows a lack of due consideration of the surroundings of a patient. While the legitimate field of surgery is the proper use of means of relief for organic or structural disorders, there are prerequisites in the recognition of the conditions warranting an operation and in the preparation of the patient for undergoing it safely, which should characterize the highest type of the surgeon.

The distinction of external and internal treatment is held by most European authorities as the basis of recognition for the practice of surgery and medicine. But this line of distinction does not imply that all disorders of the inner structures are to be left for the treatment of the practitioners of medicine. Nor should all the pathological conditions not demanding operations be excluded from the domain of surgery. A proper recognition of the scope of surgery and medicine is that of organic and functional disorders of the system.

With this limitation of the province of the physician as contradistinguished from that of the surgeon, the work of the former should be confined to such measures as are calculated to correct the performance of the functions of the different organs of the body whether internal or external.

When organic changes ensue, whether demanding the use of medication or a resort to operative measures, the case comes then within the field of surgery. It presents a modification of structures, more or less pronounced, which alters the constituents so as to produce a departure from the normal state of the part involved, in its size, shape, or density. An incision or a puncture may be requisite to diagnosticate such a condition, but if this change of structure could be clearly ascertained without any exploratory operation, the case would still fall within the domain of surgery ; with this distinction between functional and organic disorders for the practice of medicine and surgery, so soon as a change in the state of the case is recognized by the physician, it should be transferred to the charge of a surgeon, and he will still have the disadvantage of combating the latter stages of organic disorders. It may be that a skillful operator is not qualified for the highest attainment in surgery, from lack of proper precaution in proceeding with an operation.

The aim of the surgeon should be a due comprehension of abnormality in the structures of the part involved, and his end be to afford relief with the least possible injury to the organ or member which is the seat of disorder. Conservative surgery may be destructive of certain parts for the purpose of saving the structures, and the properly qualified surgeon should consider maturely every phase of the case under examination, so as to assume the responsibility of lopping off the disease and prevent its extension to the sound tissues. He who fails to use the knife or the cautery, when a resort to either would stay the progress of disease, is not the exponent of conservative surgery, but, on the contrary, aids and abets the work of destruction in the parts implicated, while he contributes ultimately to the death of the patient.

Ignorance and inexperience often lead to sad results in meddlesome surgery, when limbs are sacrificed or organs mutilated, to gratify the desire to figure as a bold operator on the part of a would-be surgeon.

In such cases no high-toned member of the profession should shield the culprit from the charge of malpractice or from the assessment of damages by a court of justice. While the allegation of malpractice, without sufficient cause, should never be encouraged by the better class of practitioners, it would be well to bring home to those who rush into the surgical arena, unknowing and unknown, the consequences of their rashness. Of the two evils over-caution against doing harm is far preferable to meddlesome surgery, and yet we would not hold him guiltless who stands with folded arms and suffers a patient to die who might be saved by a timely operation.

There is a field in surgery for masterly inactivity, and non-interference is to be highly commended when by an operation a fatal result is precipitated. The want of a proper investigation of a case presenting grave complication may lead a physician to delay in calling a surgeon until the opportunity for relief has passed and his interference can avail nothing, so that he is justified in standing aloof.

There are numerous instances of the failure in surgical procedure from the late period of performing an operation, and it is greatly to be desired that a surgeon should be called in proper time by physicians having cases under their care which are likely to require surgical interference. If a consultation should be called and the surgeon found no indications for an operation, then of course the physician would be relieved of any future responsibility in proceeding with the treatment. But most probably their joint attendance would lead to the most satisfactory result in enabling them to determine upon the conditions developed in the progress of the case which might warrant an operation.

The dilatory spirit manifested by patients and those around them, as to resisting surgical means of relief in acute cases, has proved a barrier to the adoption of the expressed views of surgeons in favor of prompt action in many cases of great urgency. All practitioners of considerable experience have had occasion to regret that the opportunity for forestalling a grave malady has been lost by the indisposition to submit to a timely operation, and they led a forlorn hope to end in disaster.

In this connection, however, it may be appropriate to consider the claims of a different case to our attention; when death is inevitable without an operation, and the knife affords the only chance, however slim that may be, for escape, it is a fair mode of dealing with this class of cases, for the surgeon to put himself in the place of the patient and determine what he would desire for himself under similar circumstances. Most of us, I think, in the possession of our faculties, would avail ourselves of the operation, and hope for the best result of it. Viewing matters from this standpoint, if those interested in the patient, manifest a desire to take the risk of which all are apprised, I think we are warranted in operating even should the probabilities be greatly against a successful result.

It is very true that untoward results, even when expected, tend to discredit surgery and to give an excuse to others for declining an operation at a certain stage when it might serve a good purpose. But considering the surroundings of the individual alone, we have certain death without an operation staring us in the face; and if the patient, after being advised of the situation, desires an operation, it should be performed.

But we have a serious question for settlement among ourselves as surgeons, and I am more especially concerned in the proper adjustment on this occasion of the differences in the surgical views of those who are equally entitled to think and to act in regard to surgical cases of great gravity.

In the course of consultation with other surgeons I have sometimes submitted a definite view in favor of immediate operation, proceeding with a confident expectation of warding off a fatal result, when the response of my colleagues has been such as to discourage any interference. On some of these occasions the unfavorable verdict has been accompanied with the statement, that if I thought proper, to take the responsibility of operation, that they would render any assistance which might be desired, without, however expecting any benefit. Of course I declined to rush into the breach under such untoward circumstances; and yet it has occurred to me that there ought to be some expression from the profession as to the line of duty for one who feels prompted to undertake the rescue of a patient from a most perilous situation, in which there is absolutely no hope without a surgical operation.

At the risk of being considered heterodoxical I would draw the attention of the profession to a consideration of the propriety of immediate operation in that desperate class of cases which result from the crushing and mangling of limbs, attended with profound shock. It has been the custom of most surgeons to watch and wait while means are resorted to for restitution of the vital forces by stimulants. With our deficient knowledge of the exact etiological factor in this anomalous condition, I am inclined to the view that a continuous baleful influence is propagated to the nerve centers from the disintegration of the structures involved, and that this may be modified favorably by a clean incision through sound tissues above the point of the injury very soon after such violence to the parts. It strikes me forcibly that surgical relief within fifteen or twenty minutes after an accident, involving the muscles, bones, nerves and blood vessels, cannot intensify the shock, and that many of the cases left to die without any operation might be rescued by a prompt amputation of the member. The A. C. E. mixture offers the most favorable conditions for an anesthetic and tends to lessen rather than increase the prostration, while the operation should be done with all possible dispatch.

It may prove the most conservative surgery to lop off structures whose vitality is completely destroyed and under the proposed state of the parts there can be no prospect of any restoration of nerve power or circulation to the tissues by delay in undertaking an operation. A proper appreciation of the participation in shock by the ganglionic nervous system should impress the surgeon with the great importance of arresting the morbific influence at the very earliest period practicable by removing the cause while energetic correctives are employed.

At this point it may be appropriate to advert to the precautions requisite in all grave operative procedures to avert a depressing effect, independent of previous violence.

I have been convinced, by observation that the use of moderate doses of quinine and strychnine during twenty-four hours, preceding any important surgical operation, serves to ward off the nervous prostration. It has also been my custom to administer an alcoholic stimulant with a hypodermic of morphine and atropia within a half hour preceding such an operation, and these preliminary measures have been attended with most satisfactory results. Prevention of shock is preferable to combating it by energetic means after a surgical operation.

As a fitting conclusion of this whole matter I would suggest that no surgical measure should be resorted to without looking into and correcting all underlying departures of the physical organization from the standard of health. It may not be possible to restore the normal condition of the secretions and excretions, yet means should be used for their correction, so far as may be practicable as a preliminary step for even the most simple operation; as the surgeon never can know in advance what complications may be developed in the course of treatment.

To prepare a patient for undergoing any capital operation, when the forces of the vital organism have been exhausted by long suffering, it is requisite to support the system by tonics and nutritious food for days or weeks prior to the surgical procedure. A neglect of this precaution is inexcusable, except in cases of urgency, when the delay would be likely to aggravate the malady.

The after treatment in surgical cases is in like manner of great importance to secure a good result, and this should include not only medication and food, but proper seclusion from exciting associations and due regard to the hygienic surroundings.

